# Acute Adrenal Insufficiency Associated with Tuberculous Vertebral Osteomyelitis and Lymphadenopathy: Case Report

**DOI:** 10.1155/2012/574845

**Published:** 2012-05-09

**Authors:** İnan Anaforoğlu, Ekrem Algün, Ömer İnceçayır, Çiğdem Şiviloğlu, İsmail Caymaz

**Affiliations:** ^1^Department of Endocrinology and Metabolism, Trabzon Numune Education and Research Hospital, 61000 Trabzon, Turkey; ^2^Department of Pathology, Trabzon Numune Education and Research Hospital, 61000 Trabzon, Turkey; ^3^Department of Radiology, Trabzon Numune Education and Research Hospital, 61000 Trabzon, Turkey

## Abstract

A 51-year-old man developed anorexia, dizziness, nausea, vomiting, and weight loss. He had orthostatic hypotension, hyponatremia, hyperkalemia, and hypocortisolemia, and the diagnosis of adrenal insufficiency was made. Magnetic resonance imaging (MRI) showed asymmetrically enlarged adrenal glands. Biopsy of a hypoechoic, enlarged, inguinal lymph node showed caseating granulomas. Lumbar MRI showed vertebral body height loss and abnormal signal in L1 and L2; vertebral biopsy showed chronic, necrotic, caseating granulomatous inflammation consistent with tuberculous osteomyelitis. Clinical improvement occurred with isoniazid, rifampicin, pyrazinamide, and corticosteroids. The differential diagnosis of adrenal insufficiency should include tuberculosis, especially in geographic regions where tuberculosis is endemic.

## 1. Introduction

After Thomas Addison described cases of adrenal insufficiency, autopsy of all 11 cases he diagnosed showed a lesion in the adrenal glands, and 6 of these cases were caused by tuberculosis [1]. During the following century, tuberculosis was reported as the most common cause of adrenal insufficiency (Addison disease) [2–4].

As effective therapies for tuberculosis were developed, the frequency of adrenal insufficiency associated with tuberculosis decreased in Western countries, and autoimmune disorders became the most common cause of adrenal insufficiency [5]. Nevertheless, adrenal tuberculosis, usually caused by hematogenous spread of pulmonary tuberculosis, continues to be a cause of adrenal insufficiency in developing countries [6, 7]. The diagnosis of adrenal tuberculosis, especially with enlargement of adrenal glands, can be difficult and may require differentiation from neoplastic disease and fungal infection [7].

Tuberculosis also may cause vertebral osteomyelitis, but it is a rare form and surgery may be needed. We report the case history of a patient who had adrenal insufficiency, vertebral osteomyelitis, and inguinal lymphadenitis caused by tuberculosis.

## 2. Case Presentation

A 51-year-old man who lived in Trabzon, Turkey, was admitted to the hospital because of anorexia, dizziness, nausea, vomiting, diarrhea, and weight loss. During the previous few months, he had developed dark skin pigmentation noted by his family and lumbar back pain. He had knee surgery for a torn meniscus 5 months before admission; the past medical history otherwise was negative. Examination showed low blood pressure (90/50 mm Hg), severe orthostatic hypotension, and inguinal lymphadenopathy. Laboratory studies showed high erythrocyte sedimentation rate (50 mm/h), high C-reactive protein (14.5 mg/L), low-serum sodium (121 mmol/L), high-serum potassium (7.5 mmol/L), high-serum creatinine (2.2 mg/dL), and low-serum cortisol (5 *μ*g/dL).

The diagnosis of acute adrenal crisis was made, and the patient was started on intravenous fluid replacement and methylprednisolone. Microscopic examination of the stool showed leukocytes and erythrocytes, and ciprofloxacin was started; however, stool cultures showed no pathologic microorganisms and the diarrhea resolved within 2 days. After 2 days of intravenous steroids, the serum sodium and creatinine levels became normal. Fludrocortisone was added because of resistant hyperkalemia.

**Figure 1:**
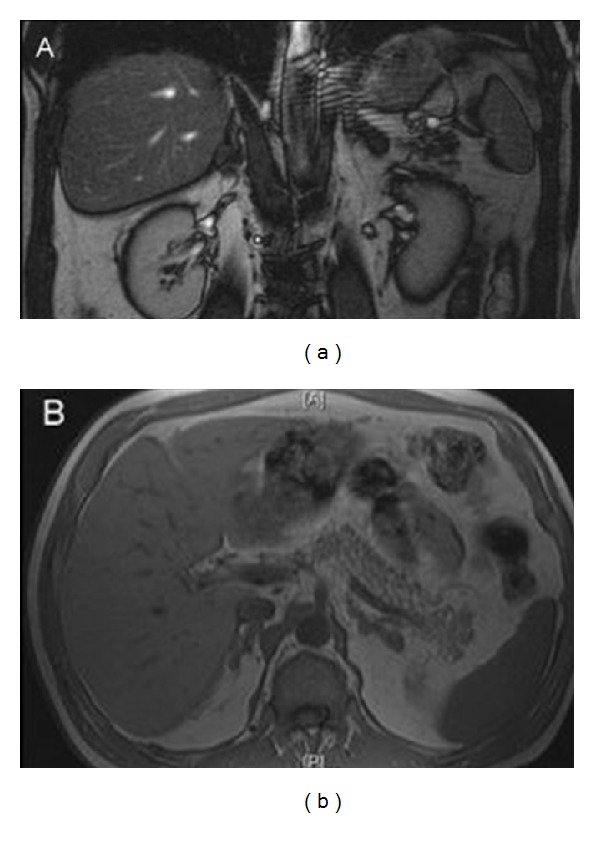
Magnetic resonance imaging of the upper abdomen showing asymmetric, bilateral enlargement of the adrenal glands.

Further studies showed a low adrenocorticotropic hormone (ACTH) level (5.41 pg/mL) under treatment. Magnetic resonance imaging (MRI) of the upper abdomen showed asymmetrically enlarged adrenal glands (coronal diameter: right, 9 mm; left, 11 mm), consistent with bilateral adrenal hyperplasia (Figure 1). Possible reasons that may cause adrenal insufficiency by infiltrating adrenal glands were sought throughly. Considering tuberculosis; the patient could not produce a sputum sample, and chest radiography was normal. 

**Figure 2:**
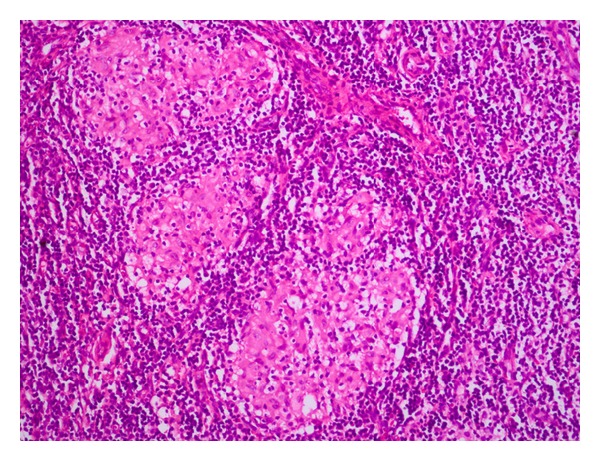
Photomicrograph of lymph node biopsy specimen showing caseating granulomas consistent with tuberculosis (hematoxylin and eosin stain; 20 fold magnification).

**Figure 3:**
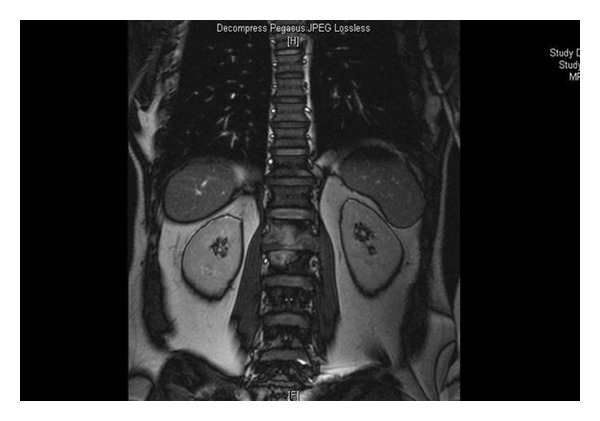
Magnetic resonance imaging of pathologic signalization in lumbar vertebrae.

**Figure 4:**
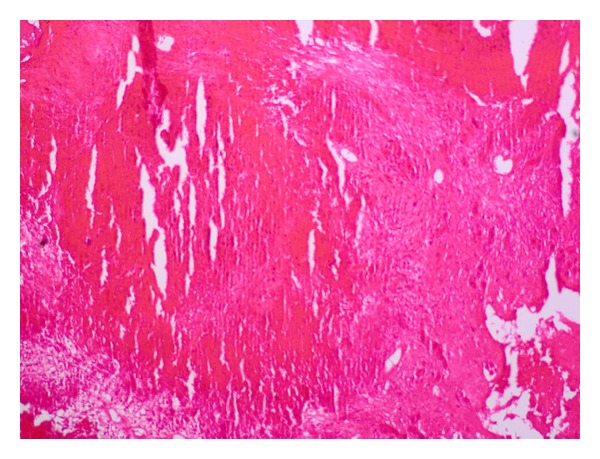
Photomicrograph of vertebral bone biopsy specimen showing caseating granulomas consistent with tuberculosis (hematoxylin and eosin stain; 20 fold magnification).

 Inguinal sonography showed multiple, enlarged (diameter, 1.5 to 2 cm), hypoechoic, nonreactive right inguinal lymph nodes with thickened cortex; excisional biopsy of an inguinal lymph node showed caseating granulomas, consistent with granulomatous lymphadenitis (Figure 2). Lumbar MRI showed loss of vertebral body height and the presence of pathologic signal in L1 and L2, consistent with compression fracture, metastasis, or infection (Figure 3); lumbar vertebral biopsy, performed by an open surgery, showed chronic, necrotic, caseating granulomas consistent with tuberculous osteomyelitis (Figure 4). Polymerase chain reaction assays of homogenates of the lymph node and vertebral specimens were negative for *Mycobacterium tuberculosis*. Further evaluation was negative for other chronic diseases, diabetes mellitus, human immunodeficiency virus infection, and malignancy.

The patient was treated with isoniazid, rifampicin, and pyrazinamide for 1 year, in addition to steroids (5 mg/day, fludrocortisone 0.1 mg/day). Two months after antituberculous therapy was completed, the glucocorticoid and mineralocorticoid drugs were stopped, and the patient was monitored closely as an inpatient. After 1 week of discontinuing steroid therapy, the morning serum cortisol was (7.3–9 *μ*g/dL), and ACTH was high (874–665 pg/mL). A Synacthen stimulation test was not performed as the level of ACTH was already very high. MRI of the adrenal glands showed bilateral asymmetric adrenal hyperplasia, similar to that observed 1 year earlier. The patient was begun again prednisolone (5 mg/d) and advised to increase the dosage during stressful conditions including fever, surgery, dental extraction, strenuous exercise with sweating, during extremely hot weather, and with gastrointestinal upsets such as diarrhea [8].

## 3. Discussion

This patient had extrapulmonary tuberculosis, with documented involvement of the lymph nodes and vertebrae, resulting in adrenal insufficiency and vertebral body collapse. Back pain had been present for several months prior to presentation, and this may be evidence that hematogenous spread to the adrenal glands occurred from the lumbar vertebrae. Most cases of adrenal tuberculosis are diagnosed 10 to 15 years after the initial infection, and most (>90%) adrenal glands are destroyed by tuberculosis during this period, resulting in adrenal insufficiency [7, 9]. It is unknown when this patient was exposed to tuberculosis.

Tuberculosis infection frequently begins at the lungs and may disseminate by the hematogenous route to extrapulmonary sites, especially organs with high blood flow such as the spleen, liver, bone marrow, kidneys, and adrenal glands [10]. Dissemination of *M. tuberculosis* may occur at the time of primary pulmonary infection or later from reinfection or reactivation of previous infection [11]. The characteristic granuloma may be caused by acute lymphohematogenous dissemination (soft or exudative granuloma, frequently having acid fast bacilli) or discharge of bacilli into microscopic blood vessels within the caseous lesions (hard granuloma, frequently having no acid fast bacilli) [10]. In the present case, no acid fast bacilli were detected from the granulomas in both the vertebral bone and lymph nodes. The spine is the most common part of the skeleton affected by tuberculosis, most commonly at the lower thoracic and lumbar vertebrae [11].

Enlargement of both adrenal glands may occur in most (90%) patients with tuberculous adrenal insufficiency [12, 13]. The imaging findings may vary with the stage and activity of the inflammatory process. In early tuberculous adrenalitis, bilateral adrenal enlargement is the typical finding, as in the present case, and this may include a central necrotic area of low attenuation and a peripheral enhancing rim [12–15]. At the late or healing stage, enlargement of tuberculous adrenals may partially or completely resolve, with or without calcification or atrophy [12–15]. No adrenal calcification was observed in the present patient. Involvement of the adrenal glands during acute (active) tuberculosis or chronic hematogenous tuberculous dissemination may cause adrenal failure. It has been stated that, adrenal enlargement may be due to activation of hypthalamo-pituitary-adrenal axis during active pulmonary tuberculosis, as active pulmonary tuberculosis is a stressful condition as direct involvement of adrenal glands by infection. It is not clear whether antituberculous therapy can reverse adrenal function in these patients [7]. In the present case, the patient had adrenal failure, but treatment for tuberculosis was required because 2 foci of tuberculosis (bone and lymph nodes) were identified other than adrenal glands.

The prognosis for recovery of adrenal function may vary in patients with tuberculous adrenal insufficiency [7, 16, 17]. Recovery of adrenal function may occur in patients treated for tuberculosis [16, 17], but absence of adrenal recovery 2 to 5 years after therapy also has been observed [9]. In the present patient, adrenal failure did not recover at short-term followup, and the patient continues to take prednisolone.

In summary, the present case shows that tuberculosis continues to be a potential cause of adrenal insufficiency. The differential diagnosis of adrenal insufficiency should include tuberculosis, especially in geographic regions where tuberculosis is endemic or in patients exposed to tuberculosis.
